# The Relation between Self-Reported Worry and Annoyance from Air and Road Traffic

**DOI:** 10.3390/ijerph120302486

**Published:** 2015-02-25

**Authors:** Frits van den Berg, Claudia Verhagen, Daan Uitenbroek

**Affiliations:** GGD Amsterdam Public Health Service, P.O. Box 2200, 1000 CE Amsterdam, The Netherlands; E-Mails: cverhagen@ggd.amsterdam.nl (C.V.); Daanuitenbroek@ggd.amsterdam.nl (D.U.)

**Keywords:** noise, odour, annoyance, worry, anxiety, aircraft noise, road traffic noise, noise survey

## Abstract

Negative perceptions such as fear or worry are known to be an important determinant of annoyance. Annoyance caused by noise and odour has been analysed in relation to worry about safety or health due to environmental hazards, using responses to a health survey. In the survey area high environmental impacts come from air and road traffic. The survey results show a correlation between worry due to the airport or passing aircraft and noise and odour annoyance from aircraft (correlation coefficient (c.c.) close to 0.6). For the relation between worry about a busy street and annoyance from road traffic the correlation is lower (c.c. 0.4–0.5). Worries about different situations, such as living below sea level, close to an airport, busy street or chemical industry, are highly correlated (c.c. 0.5–0.9), also for situations that are not obviously related. Personal factors can also lead to more worry: being female, above 35 years of age, having a high risk for anxiety/depression and being in bad health increase the odds for being worried. The results thus suggest that worry about safety or health is correlated to both personal and environmental factors.

## 1. Introduction

Worry about an environmental hazard can result from the perceived presence of that hazard and it can be hypothesized that a more intrusive presence will lead to a stronger reaction, *i.e.*, more worry. It is likely that the reaction will vary between individuals, depending on personal characteristics such as the sensitivity to environmental signals or hazards. Thus we expect that worry about an environmental hazard will depend on the (visual, aural and olfactory) perception of a hazardous activity and on personal factors. 

This paper seeks to explore the relationship between worry and annoyance reported in a public health survey by respondents who live close to a main airport. Worry, a “harassing anxiety” [1] is a mental process aiming to avoid unpleasant or threatening situations in the past or future [2]. Miedema and Vos have shown that feelings of fear have a significant and substantial effect on annoyance from a noise source [3]. This effect was found for road, rail and air transport. Miedema and Vos argued that the relation between fear and annoyance could either depend on the actual experience of fear or could result from a common predisposition to fear and annoyance. Communication could reduce feelings of fear and thus reduce noise annoyance, but they do not expect such an effect when a predisposition to experiencing fear is the cause of more noise annoyance, because a personal characteristic will not depend on information or communication [3]. 

In a lab study Standing and Stace measured state and trait anxiety, *i.e.*, anxiety related to a specific situation and anxiety at the usual personal level [4]. Trait anxiety differed considerably between subjects and did not depend on the noisiness of the situation. They found that in a noisy condition the increase in state anxiety with noise level was rather similar for three subgroups with different trait anxiety levels. This suggests that the anxiety level of a person is in part a personal characteristic and for another part depends on the situation. Stansfeld *et al.* [5] concluded that there was some evidence that road traffic noise could lead to an increase in anxiety, confirming the results of previous studies. 

Miedema and Vos [6] mention that (trait) anxiety and noise sensitivity are related and they argue that noise sensitivity has an important role in the generation of anxiety or worry by aircraft. Noise sensitivity, a heightened perception of environmental sounds, can explain part of the reaction to noise and is considered to be a personal trait. Noise sensitive persons are also likely to be sensitive to other aspects of the environment and noise sensitivity has been found to be associated with neuroticism [7]. Nordin *et al.* [8] found support for the hypothesis of noise sensitivity being associated with perceived stress and odour sensitivity. Stansfeld *et al.* [7] stated that noise sensitive individuals “attend more to noises, discriminate more between noises, find noises more threatening and out of their control, and react to, and adapt to noises more slowly than those who are less sensitive in this way”. Weinstein [9] concluded that being critical or uncritical in the assessment of the environment explained a significant part of the noise annoyance experienced by respondents. The tendency to be critical was associated with a higher noise sensitivity and also with a higher sensitivity to other environmental factors. There was more variation in the ratings of the different environmental aspects given by critical persons compared to ratings given by uncritical persons, suggesting critical persons were not generally more negative but more discriminating then uncritical persons [9].

Annoyance can also be related to a non-permanent mental state. Jonsson and Sörensen [10] showed in an experimental field study that influencing subjects to adopt a positive attitude towards aviation reduced their annoyance from aircraft noise at home. Similarly in a lab study they found a reduction in annoyance from aircraft and motor traffic noise. Maris *et al.* [11] showed in a lab study that fair treatment influences annoyance. Part of the participants could give their preference for sound samples to be played when doing a task. Although in fact the same sample was presented to all participants, the results showed that annoyance ratings were significantly lower when a preference could be given compared to the situation no preference was asked. Munz *et al.* [12] manipulated participants’ stress level and found that when a sound interfered with a laboratory task this raised the stress level, but not when the same sound was congruent with the task. 

The main aim of the present study is to gain more insight in the relation between worry about a noise source and annoyance from that source. This is of interest for the GGD Amsterdam Public Health Service, as flight routes to and from Schiphol Amsterdam Airport are a major noise source in the working area of the GGD. The high prevalence of noise annoyance from air traffic has been important in the local and national political agenda for years. In recent years several measures have been taken to reduce the noise exposure, such as steep ascents, quiet (low) descents and strict route keeping. Reduction of fear or worry could also be relevant as a possible route to less noise annoyance. It is therefore important to understand whether the negative emotional association is related to the actual noise or to personal characteristics. Road traffic is included in this analysis as a general noise source to compare it with the more local air traffic. 

Thus, two topics are of primary interest: the questions of whether individual worries are connected to the environmental impact of aircraft, and if being worried is associated with personal characteristics. 

## 2. Methods

### 2.1. Study Design and Study Sample

**Figure 1:**
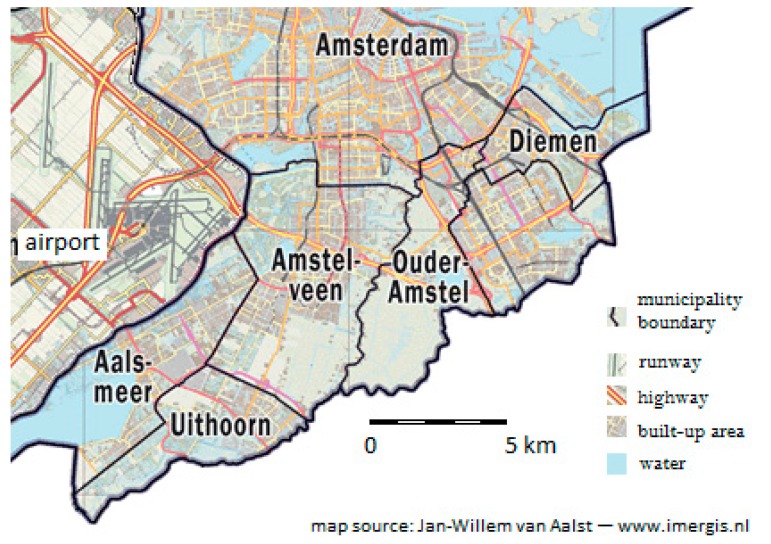
Study area with five surveyed municipalities, airport (grey area with runways) at west and Amsterdam at north.

To support public health policy, regional and local Dutch Public Health Services (PHS, in Dutch: GGD) regularly survey the status of health and health-related topics of the population in their area. Here results are used from the survey held in 2010 in five municipalities (Aalsmeer, Amstelveen, Ouder-Amstel, Uithoorn and Diemen) directly south of Amsterdam, denoted here as Amstelland (or AL). Together with Amsterdam they form the working area of GGD Amsterdam. Figure 1 shows a map of the municipalities and the airport. 

Most of the inhabitants live in urban area contiguous with the city of Amsterdam. The western border of the area is next to Amsterdam Airport Schiphol processing over 50 million passengers, 1.5 million tons of cargo and 400,000 aircraft movements per year. The population in the AL area comprises 173,000 people, of which 51.6% are female. 21% are 18 years of age or younger, 62% are between 18 and 65 years, and 17% are 65 years or older. From those above 18 years 6876 persons, evenly distributed over five age groups (19–34, 35–49, 5–64, 6–74, 75+) and randomly selected from the population administrations, were asked to take part in the survey. Of these, 3817 persons (response rate 55.5%) completed the paper questionnaire or an online questionnaire. Questions on perceived risk and annoyance were not presented to the oldest age groups, but only to the 19–64 age groups, including 1,968 respondents. There was no non-response test. Respondents as a group did not deviate substantially from the Dutch population with respect to ethnicity, marital or employment status. No national data were available on education level that could be compared to the study group [13].

### 2.2. Questionnaire

In the survey standard questions on 34 health indicators related to topics such as smoking, physical activity, psychosocial problems or health care use. In the survey a question on the perceived risk of environmental factors was included, as well as questions on noise and odour annoyance and on perceived sleep disturbance from noise. This allows investigating the relation between the perceived risk from aircraft routes, the airport and other sources, and annoyance from these sources. Relations between annoyance and self-reported sleep disturbance have been investigated separately [14]. The question about perceived risk to health/safety was formulated as follows:

*Which of the following situation applies to you? Please tick these and then indicate whether you are worried about your safety/health in this situation. You can indicate this by ticking a number from 0 through 10, where 0 means you are not annoyed at all and 10 that you are extremely annoyed.*

The items to answer were as follows: I live… in a busy street/in a polder (*i.e.*, land under the level of the sea or close to a river)/close to a (petro)chemical plant/close to an airport/under the air route of a main airport/in an agricultural area/close to power lines/close to broadcasting stations for radio and TV or antennae for mobile telephones (GSM base station)/along a route (road, water, rail, pipe) for dangerous goods/on polluted ground/close to a petrol station/close to a nightlife district/close to a hazardous business (such as a fireworks factory). First, the respondent was to tick a box whether the situation applied to his or her situation. If so, the degree of worry was scored on an eleven point scale (0…10). The underlined items (in this question and the questions below) are the main items in the present paper.

In the Dutch question the term for worried (‘bezorgd’) denotes there is or may be a hazard with respect to the situation involved. The Dutch term is ambiguous as it can be interpreted as actually being worried or as a judgment that there is a risk (“being concerned” might be a better translation for the latter interpretation), without there being necessarily an implication of “brooding” over the situation—as is more often implied with the English term “worried”. In analogy with annoyance sources, the situations that may lead to worry (as listed above) will also be denoted as “worry sources”. The question about annoyance from noise complies with ISO/TS 15666:2003 and was formulated as follows:

*Below is a scale from 0 to 10 where you can indicate to what degree you are annoyed, disturbed or irritated when you are at home. If you are not annoyed at all, please tick a 0, if you are extremely annoyed, please tick a 10. If you are somewhere in between, please tick a number between 0 and 10. If the sound is not present in your home, you can tick this in the first column.*

*Thinking about the past 12 months, which number from 0 to 10 indicates best to what degree you are annoyed, disturbed or irritated by sound from sources listed below when you are at home? Please put a tick on every line.*

The list of noise sources was: traffic on roads with a speed limit higher than 50 km/h/traffic on roads with a speed limit of 50 km/h or less/trains/trams (urban or regional) or metro/aircraft/Schiphol airport (taxiing, engine testing and/or other ground activities)/businesses or industry/neighbours/scooters or mopeds/other. The respondent could tick a separate box labelled ‘Not audible’ preceding the eleven point scale. The underlined items will further on be abridged to city traffic/aircraft/airport.

The question about annoyance from odour was the same question as for noise, with odour instead of noise. The list of odour sources was: road traffic/aircraft/businesses or industry/agricultural and cattle breeding activities/hearth or multi-burner in the neighbourhood/other. The respondent could tick a separate box labelled “Not noticeable” preceding the eleven point scale. 

The response on the questions can be stratified for a number of individual characteristics addressed in the survey. Characteristics relevant for this analysis, each stratified into categories (between brackets), are:

- year of birth (age in three categories: 19–34, 35–49, 50–64 years)
- gender (male, female)
- perceived health (categories: excellent/good, moderate/bad)
- risk to suffer from anxiety/depression (categories: high = total score 15–30, low = total score 10–15)
- feeling lonely (categories: high = score 9–11, low = score 0–8)

The survey questions pertaining to anxiety/depression are based on Kessler *et al.* [15], the question about social contacts (feeling lonely) are based on De Jong Gierveld and Kamphuis [16]. 

### 2.3. Statistical Analysis

The focus of this study is the relation between the response on the worry and annoyance questions in relation to aircraft. Road traffic is included because it is an important environmental presence in urban areas and may serve as a reference for environmental impact. Sources of worry and of annoyance are described in similar, but not always identical words in the questionnaire. When correlating scores for worry with scores for annoyance, the worry source ‘air route’ will be compared to the noise annoyance source “aircraft” or to the odour source “aircraft”. The worry source “airport” will be compared to the noise annoyance source “airport” or to the odour source “aircraft”. Finally, the worry source “busy street” will be compared to the noise annoyance source “city traffic” or to the odour source “road traffic”. It is not clear whether respondents would associate a busy street also with highway traffic, trams or scooters/mopeds which are all listed as noise annoyance sources and these sources are therefore excluded.

The terms “highly worried” or “highly annoyed” imply a score of 8, 9 or 10. This is similar to the classifications given by Schultz [17] (upper three scales of eleven-point scale or upper two of seven-point scale) and close to the classification by Miedema and Vos [18] (upper 28% of scale). Being worried or annoyed implies a score from 4 through to 7, inclusive.

Respondents who were not in a situation where they could perceive a risk to their health or safety and respondents who indicated they could not hear a noise source or notice an odour are mentioned as part of the study population but were not included in the analysis. Correlations are expressed as Pearson’s correlation coefficients. The relation with respondents’ characteristics was examined using Chi-squared tests. Statistical significance is assumed at *p* < 0.05 and shown in the figures. Linear regression was used to test the relationship between annoyance and worry for various sources. Significance of the slope was determined by using the t-test. Lastly, data were weighted by a number of demographic and psychological factors to ensure that the results of this study are representative for the population concerned.

### 2.4. Aircraft Noise Sound Levels

The geographical position of respondents’ homes is known only as a 4-position postcode area. The size of these areas vary, but in the Netherlands each on average contains 2000 households. There are 21 4-position postcode areas in the study area. Aircraft sound levels are calculated as L_den_ values from aircraft routes, speeds and motor settings according to the Dutch legal instructions [19]. L_den_ levels were calculated at each grid point in a 250 m by 250 m grid and the level at a residential address was taken from the nearest grid point. L_den_ levels for each postcode area were calculated as the average of all residential addresses in the postcode area. 

## 3. Results

### 3.1. Determinants of Reported Worry Related to Air Route and Airport 

**Figure 2:**
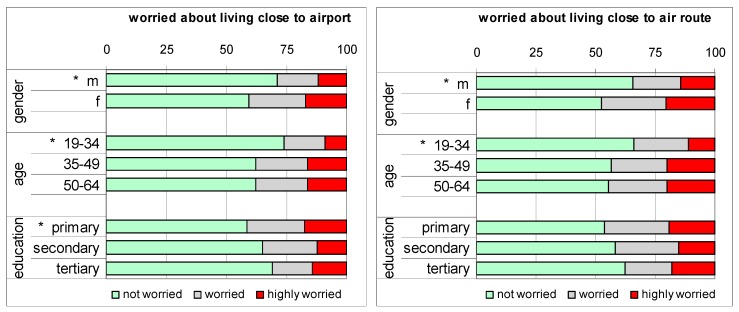
Percentages of respondents that are not, somewhat or highly worried about living close to airport (left panel) or close to an air route (right panel), stratified by gender, age group and education level. * indicates significant difference (*p* < 0.05).

About two-thirds (1291) of the 1968 respondents aged 19–64 indicated they live close to the airport and 1202 live under an air route to the airport. Of those living close to the airport or an aircraft route 37% and 42%, respectively, are worried. Scores for worry related to the airport and scores for worry related to an air route are highly correlated (correlation coefficient 0.94). There are significant differences in the percentages of worried respondents with respect to gender and age (see Figure 2): men and younger persons are less worried (*p* < 0.05). People with lower education are more worried than those with higher education, but this difference is statistically significant only for those reporting to live close to the airport. 

**Figure 3:**
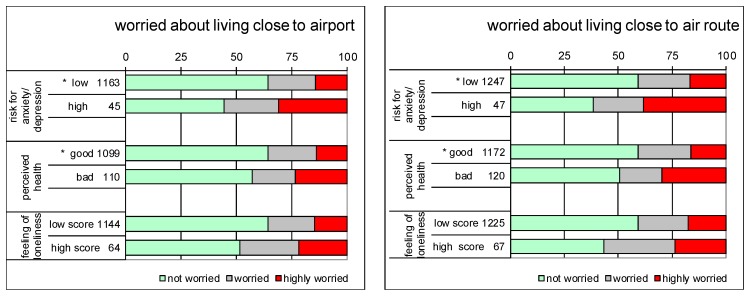
Percentages of respondents that are not, somewhat or highly worried about living close to airport (left panel) or close to air route (right panel), depending on risk for anxiety/depression, perceived health and feelings of loneliness. Numbers are number of respondents. * indicates significant difference (*p* < 0.05).

The numbers of respondents having a high risk for either anxiety/depression, bad health or feeling lonely each amount to 4% to 10% of all respondents. Figure 3 shows how these personal factors are associated to worry about living close to the airport and an air route. Respondents with a high risk of anxiety/depression are significantly more likely to be highly worried about living close to the airport or an air route compared to those with a low risk (all *p* < 0.05). Also, respondents who report to have bad/moderate health are significantly more likely to be highly worried about living close to the airport or an air route compared to those with good/excellent health. Respondents with a high score for feeling lonely are not significantly more likely to be worried compared to those with a low score.

### 3.2. Worry and Annoyance

One-fifth (376) of the 1968 respondents reported to live in a busy street; 48% of these 376 respondents are worried. Other environmental hazards (from the list in Section 2.2) lead to lower numbers of worried respondents, either because the situation presents itself less often or respondents perceive a lower risk. Table 1 shows that the fraction of respondents who report to live close to the airport, an air route or on a busy road and are worried is 36% to 48%; 10% to 12% are highly worried. Highest percentages of annoyance are associated with noise from air routes (64%), lower percentages are found for road traffic noise (30%–49%) and airport noise (17%). For annoyance from odour the percentages of annoyed respondents are smaller and percentages are more similar (17%–19%).

**Table 1:** Number of respondents (N) that report to live in a specific situation and can be annoyed by noise or odour from a source related to that situation; %(H)W = percentage (highly) worried, %(H)A = percentage (highly) annoyed, all as fractions of N (numbers differ slightly because of missing values).

Respondents Living…	N	%W	%HW	Annoyed by…	%A	%HA
close to air route	1291	42	11	air route noise	64	26
	1286	42	11	aircraft odour	17	6
close to airport	1193	36	10	airport noise	17	5
	1202	37	10	aircraft odour	17	5
on busy road	375	47	12	city traffic noise	49	16
	373	46	12	highway noise	30	8
	376	48	12	road traffic odour	19	5

**Figure 4:**
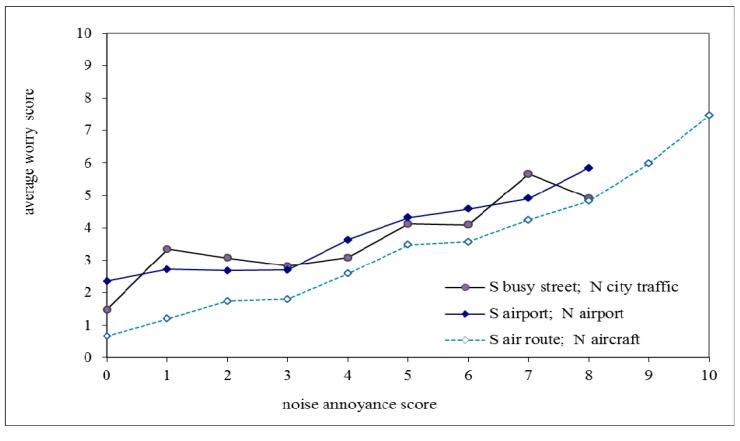
Relation between average worry score and **noise** annoyance score for respondents living in one of three situations (close to aircraft route, close to airport and on busy street) and who report to hear the noise source. Legend: S = situation (living on/close to…); N = noise source.

**Figure 5:**
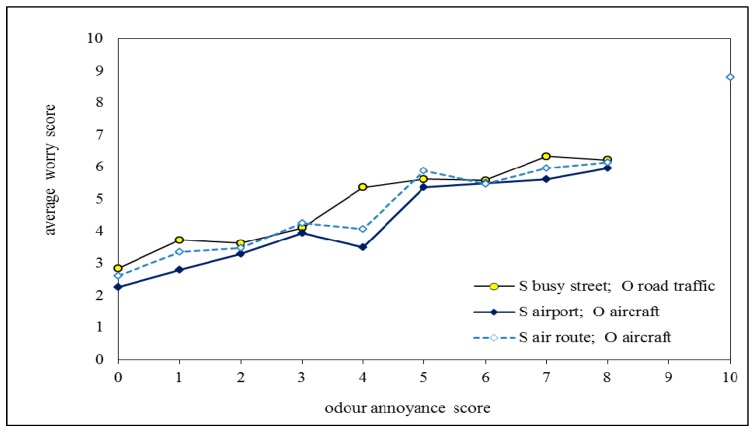
Relation between average worry score and **odour** annoyance score for respondents living in one of three situations (close to aircraft route, close to airport and on busy street) and who report to smell the odour source. Legend: S = situation (living on/close to…); O = odour source.

In Figure 4 the average worry scores related to living close to an air route, airport or busy street are plotted as a function of the annoyance scores related to aircraft, airport and road traffic noise, respectively. Data points based on less than 20 respondents are omitted. Figure 4 shows that at the same annoyance score the worry score associated with an air route is lower than for the airport; both worry scores are also highly correlated (correlation coefficient is 0.94). For road traffic the average worry score is plotted *versus* annoyance from city traffic noise. The average worry scores *versus* odour annoyance scores are plotted in Figure 5. 

The worry scores in Figure 4 increase on average with 0.5 ± 0.1 units per unit of annoyance score for aircraft and airport noise (slope of best linear fit to lines plotted in Figure 4 is 0.62 and 0.43, respectively) and 0.40 for city traffic noise. In the case of odour annoyance (Figure 5) the slope is 0.5 ± 0.05 units per unit of annoyance score (0.55 and 0.48 for aircraft odour in relation to living close to an air route and airport, and 0.45 for road traffic odour). 

Figure 4 shows that at zero annoyance, where respondents report that they do hear the source, the average worry score is positive. This average score is 2.3 for those who report to live on a busy street, 1.5 for those close to the airport and 0.7 for those close to an air route. We can compare this to respondents who report to live in the same situation but do not hear the source. Then the average worry score is, in the same order, 2.7 (with 36 respondents who do not hear road traffic noise out of 375 that live on a busy street), 2.7 (653 who do not hear the airport out of 1193 that live close to the airport) and 0.8 (only 23 who do not hear aircraft out of 1291 that live close to an air route). 

Table 2 shows the correlation coefficients for relations between the worry and annoyance sources. Only respondents who reported living in the situation at hand are included. When the annoyance source corresponds to the situation of worry (such as annoyance from road traffic and living in a busy street) sources are considered to be related. When the annoyance and worry sources do not correspond (such as annoyance from road traffic and living close to an air route) sources are considered to be unrelated. The coefficients for the correlation between noise or odour annoyance with worry about related sources in Table 2 are 0.43–0.63, for the unrelated sources this is 0.27–0.40. Noise annoyance and odour annoyance are also correlated. Correlation coefficients between both annoyances for sources in Table 2 range from 0.53 to 0.59.

**Table 2:** Pearson’s correlation coefficients between individual scores for worry with respect to a situation and scores for annoyance from noise or odour from related (bold) and unrelated (italics) sources. All coefficients are significant at *p* < 0.01.

Worried About…	Noise Annoyance from			
	Air Route	Airport	City Traffic
living close to air route	**0.63**	0.42	*0.31*
living close to airport	0.59	**0.43**	*0.29*
living on a busy street	*0.27*	*0.28*	**0.49**
	**Odour Annoyance from**		
	**Aircraft**		**Road Traffic**
living close to air route	**0.56**		*0.31*
living close to airport	**0.57**		*0.35*
living on a busy street	*0.40*		**0.46**

We further exploited the relation between worry and annoyance in a multiple linear regression. Keeping constant for demographic and psychological factors hardly changed the relation between worry and annoyance. When keeping these factors (gender, age, education, risk for anxiety, perceived health and feelings of loneliness) constant, the average increase in worry for each point increase in annoyance changed with less than 0.01 for aircraft, airport and road traffic, or less than 0.1 over the entire range of annoyance scores. The correlation coefficients for these corrected worry-annoyance relations, based on individual scores, are all three highly significant (*p* = 0.000).

### 3.3. Correlations between Worry Scores 

If worry about health or safety is determined by personal factors, one would expect that when people are exposed to a number of environmental hazards, the worry scores for the different situations depend on the personal factors and thus are correlated. This has been investigated by calculating the correlation coefficients for all possible binary relations between scores for the thirteen sources of worry mentioned in the questionnaire (see Section 2.2). All 78 possible pairs of worry scores are significantly correlated (two-sided, *p* < 0.01). Correlation coefficients for nearly all (76) relations are ≥0.45 and for 38 even ≥0.60. Some of the worry sources in these relations may have a common basis which makes a correlation plausible. E.g., a (petro)chemical plant or petrol station might both be perceived as a hazardous business, or the petrol station may be mentally connected to a route for dangerous goods; power lines may well be conceived of as electromagnetic sources, similar to broadcasting stations or antennae. But often there is no obvious connection between the hazards of different situations, e.g., living beneath sea level or in an agricultural area or below an air route. Correlation coefficient for unrelated pairs are not clearly lower than for related pairs. 

There is no indication that respondents give higher worry scores when living in a larger number of situations. The average of the worry scores for all respondents who report to live in the same number of situations is 2.5 ± 0.25, independent of the number of situations that range from one up to seven (seven being the highest number of situations that respondents report to live in).

### 3.4. Exposure to Aircraft Sound

**Figure 6:**
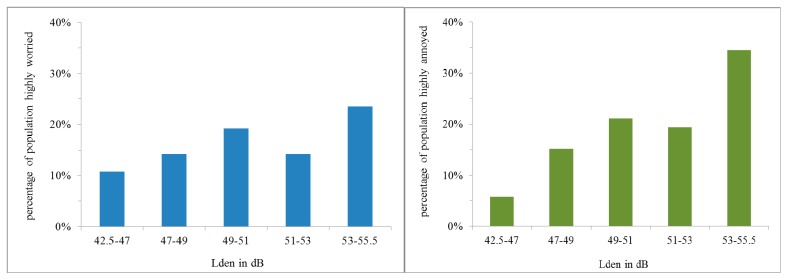
Left: percentages of population highly annoyed by aircraft noise in five categories of aircraft noise level L_den_. Right: percentages of respondents that are highly worried about living close to an air route

L_den_ levels per postcode area ranged from 42.5 to 55.5 dB. These have been grouped in five classes of aircraft sound level as indicated in Figure 6. The data were weighted to be representative for the Amstelland population. In the left panel of Figure 6 the percentages of the population who are very annoyed with aircraft sound have been plotted. In the right panel the percentages who are very worried about their health/safety because of living close to an air route have been plotted. There is a steady increase of these percentages with sound level except around 52 dB, where relatively low percentages occur in three of the four postcode areas in this sound level class. The three areas lie in the extreme southwest of the study area, but we have no explanation for the lower percentages here. 

## 4. Discussion 

The results show there is a strong correlation between annoyance from aircraft or airport noise and worry about the risk for health and/or safety associated with living close to an air route or airport. Worry from living in a busy street and noise and odour annoyance from road traffic are also significantly correlated. These results concur with those from Miedema and Vos [3]: fear or a perception of risk is an important factor in relation to noise annoyance. Annoyance from odour was also highly correlated to worry as has been reported earlier [6,8]. 

In each situation (living close to an air route, the airport or on a busy street) the average worry score is associated with an increase in annoyance. According to the WHO annoyance can serve as a (subjective) exposure variable in statistical analyses of noise and health end points [20]. Our results show that, for aircraft noise, worry increases with both the subjective exposure (annoyance) and the objective exposure (sound level). Thus, our results support earlier results [4,5,9] that a higher exposure is associated with a higher (state) anxiety. This is true for different environmental factors: noise and odour. Worry about health and safety risks is apparently related to both signals coming from the source of worry and though noise leads to more annoyance, the signals have approximately the same differential effect: the increase in the average worry score with noise and with odour annoyance is more or less comparable for air and road traffic. However, the worry does not vanish at zero annoyance: part of those that are not annoyed by the noise or odour still report to be worried. And respondents who do not hear a source may still report to be worried. Worry therefore occurs also when an environmental signal (noise or odour) is absent, though the presence of a (stronger) signal is associated with more worry.

In this study, male and young adult (19–34 years) respondents were significantly less worried about living close to an airport or aircraft route. That women worry more than men is well-known in psychological literature [21]. According to a recent American survey 22% of women often feel worried, nervous, or anxious compared to men 17% of men [22]. Age and gender have also been found to have an influence on risk perception, but not consistently, perhaps because they are mediated by other intervening factors such as education [23]. In the recent American survey [22] men aged 45–64 years were about as likely to have feelings of worry, nervousness, or anxiety as men aged 18–44 years (though both groups more than men ≥ 65 years). This is in contrast to the present results where younger men (19-34) were significantly less worried, but our results include worry about environmental factors only. 

Respondents with a self-perceived bad health are significantly more worried about living close to an airport or aircraft route. Feeling less healthy may make a person more vulnerable to external stressors or less able to cope with added stressors and both factors are determinants of annoyance [7]. Also respondents with a higher risk of anxiety/depression are significantly more worried. This is in line with results from Stansfeld *et al.* [5] who found an association between noise and increased anxiety scores. This also concurs with results from Muris *et al.* [24] who used standard questionnaires to investigate the correlations between rumination and worry on the one hand and neuroticism and anxiety on the other. They found significant pathways from neuroticism directly to anxiety, and indirectly via rumination and worry. Perhaps this may explain the result that respondents worrying about one situation are likely to worry about one or more other situations as the correlations between scores for worry about different situations show. 

A weakness in this study is the lack of objective correlates to the subjective perceptions at the location of each respondent. We have no objective measure of airport sound level (which is not available at all), of road traffic sound levels (which is available, but we do not know each respondent’s position with sufficient precision), or of odour intensity (which is not available at all). However, there is an estimate of aircraft sound level, based on the exposure of the population in each postcode area. Apart from statistical fluctuations related to the number of respondents per postcode area, this estimate is correct for the study group as they represent a random sample from the population. We could also have matched respondents’ perceptions of living ‘close to’ the airport from their postcode positions, but this would only be a rough estimate as the average size of a 4-position postcode area in The Netherlands is 8 km^2^. This weakness does not flaw the study as the focus is on self-reported items and thus on the perception of environmental impacts. Nevertheless, objective measures would help to understand the relation with these perceptions. 

The questionnaire was not dedicated to this study, so we had to depend on the available questions and were not able to add or change questions to refine this study. As a result the sources of worry or annoyance were not labelled identically which may have influenced the results. For example, scores for annoyance from road traffic noise could differ from scores for annoyance from a busy road. 

## 5. Conclusions 

Worry about a situation may depend on the situation at hand—living close to an airport, aircraft route or busy road—but part of this worry is apparently connected to other, even unrelated situations. This suggests that worry is, in part, a general factor related to respondents’ characteristics, similar to trait anxiety. But worry also depends on (perceived) exposure to the source of noise or odour connected to the situation. This is to some extent specific to that source, as the correlation between worry and annoyance is stronger when worry and annoyance source are related. 

Our results indicate that worry about a situation in the living environment depends on both personal and environmental factors. Worry is related to the situation, because worry and annoyance both depend on the presence of noise and/or odour. But worry and annoyance also have a personal characteristic as a common factor that is probably related to noise sensitivity. Thus, worry can occur without a perceived exposure to noise or odour, but the presence of such an exposure enhances the level of worry. This concurs with earlier results that trait anxiety is influenced by noise whereas state anxiety depends on personal factors. We conclude that more noise or odour is related to more worry, and this has more effect on persons that have a higher personal risk for being worried and annoyed. 
